# 2,6-Bis[(4*R*,5*R*)-4,5-diphenyl-4,5-di­hydro-1,3-oxazol-2-yl]pyridine

**DOI:** 10.1107/S1600536811014668

**Published:** 2011-05-07

**Authors:** Ning Lin, Yan-Qiu Deng, Miao-Miao Chen, Ren-Shi Luo, Seik Weng Ng

**Affiliations:** a Institute of Drug Synthesis and Pharmaceutical Processes, School of Pharmaceutical Sciences, Sun Yat-sen University, Guangzhou 510006, People’s Republic of China; b Department of Chemistry, University of Malaya, 50603 Kuala Lumpur, Malaysia

## Abstract

The mol­ecule of the title compound, C_35_H_27_N_3_O_2_, lies on a twofold rotation axis passing through the pyridine ring. The five-membered ring is approximately flat (r.m.s. deviation = 0.065 Å) and is essentially coplanar [dihedral angle = 4.2 (2)°] with the pyridine ring.

## Related literature

For the synthesis of the precursor, see: Desimoni *et al.* (2001 ▶). For the structure of 2,6-bis­(2-oxazolin­yl)pyridine, see: Sada *et al.* (2003 ▶).
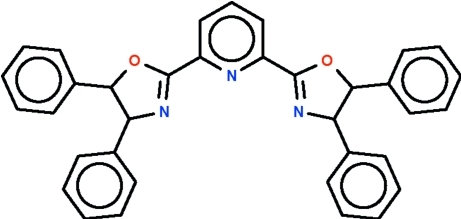


## Experimental

### 

#### Crystal data

C_35_H_27_N_3_O_2_
*M* *_r_* = 521.60
Monoclinic, 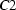

*a* = 19.035 (2) Å
*b* = 6.5908 (7) Å
*c* = 14.3001 (15) Å
β = 129.454 (1)°
*V* = 1385.2 (3) Å^3^
*Z* = 2
Mo *K*α radiation
μ = 0.08 mm^−1^
*T* = 293 K
0.38 × 0.20 × 0.16 mm

#### Data collection

Bruker SMART CCD diffractometer
3266 measured reflections
1299 independent reflections
1190 reflections with *I* > 2σ(*I*)
*R*_int_ = 0.017

#### Refinement

*R*[*F* ^2^ > 2σ(*F* ^2^)] = 0.034
*wR*(*F* ^2^) = 0.099
*S* = 1.05
1299 reflections
182 parameters
1 restraint
H-atom parameters constrained
Δρ_max_ = 0.16 e Å^−3^
Δρ_min_ = −0.11 e Å^−3^
Absolute structure: 768 Friedel pairs merged

### 

Data collection: *SMART* (Bruker, 2001 ▶); cell refinement: *SAINT* (Bruker, 2001 ▶); data reduction: *SAINT*; program(s) used to solve structure: *SHELXS97* (Sheldrick, 2008 ▶); program(s) used to refine structure: *SHELXL97* (Sheldrick, 2008 ▶); molecular graphics: *X-SEED* (Barbour, 2001 ▶); software used to prepare material for publication: *publCIF* (Westrip, 2010 ▶).

## Supplementary Material

Crystal structure: contains datablocks global, I. DOI: 10.1107/S1600536811014668/bt5522sup1.cif

Structure factors: contains datablocks I. DOI: 10.1107/S1600536811014668/bt5522Isup2.hkl

Additional supplementary materials:  crystallographic information; 3D view; checkCIF report

## Figures and Tables

**Fig. 1.**
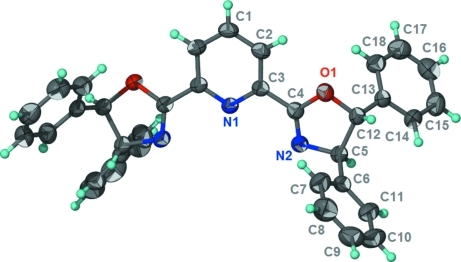
Anisotropic displacement ellipsoid plot (Barbour, 2001) of C35H37N3O2 at the 50% probability level; H atoms are drawn as arbitrary radius.

## supplementary crystallographic information

### Comment

We report here the cyclization of the two side arms of (2,6-bis[(1*R*,2*S*)*N*,*N'*-2-chloro-1,2-diphenylethyl]-pyridinedicarboxamide to form a di-substituted pyridine having two 1,3-oxazoline rings. The title compound is intended for an evaluation of its phamaceutical properties. It lies on a twofold rotation axis that passes through the pyridine ring (Fig. 1). The five-membered ring is approximately flat [r.m.s. deviation 0.065 Å] and is nearly coplanar [dihedral angle 4.2 (2)°] with the pyridine ring. In the parent compound, 2,6-bis(2-oxazolinyl)pyridine, the three rings are similarly nearly coplanar (Sada *et al.*, 2003).

### Experimental

To a suspension of 2,6-bis[(1*R*,2*S*)*N*,*N'*-2-chloro-1,2-diphenylethyl]-pyridinedicarboxamide (Desimoni *et al.*, 2001) (0.89 g, 1.5 mmol) in ethanol (26 ml), an equeous solution of 2 N sodium hydroxide (13 ml) was added and the mixture was refluxed 8 h. The hot suspension was filtered and the white solid was washed with water (0.73 g, yield 90%). Crystals were obtained by recrystallization from ethyl acetate; m.p. 474–474 K.

### Refinement

H atoms were placed in calculated positions (C—H 0.93–0.99 Å) and were included in the refinement in the riding model approximation, with *U*(H) set to 1.2*U*_eq_(C); 768 Friedel pairs were merged. The absolute configuration was assumed to be that of the reactant.

### Crystal data

**Table:**

C_35_H_27_N_3_O_2_	*F*(000) = 548
*M**_r_* = 521.60	*D*_x_ = 1.251 Mg m^−^^3^
Monoclinic, *C*2	Mo *K*α radiation, λ = 0.71073 Å
Hall symbol: C 2y	Cell parameters from 1652 reflections
*a* = 19.035 (2) Å	θ = 2.8–26.1°
*b* = 6.5908 (7) Å	µ = 0.08 mm^−^^1^
*c* = 14.3001 (15) Å	*T* = 293 K
β = 129.454 (1)°	Prism, colourless
*V* = 1385.2 (3) Å^3^	0.38 × 0.20 × 0.16 mm
*Z* = 2	

### Data collection

**Table:**

Bruker SMART CCD diffractometer	1190 reflections with *I* > 2σ(*I*)
Radiation source: fine-focus sealed tube	*R*_int_ = 0.017
graphite	θ_max_ = 25.0°, θ_min_ = 2.9°
φ and ω scans	*h* = −22→22
3266 measured reflections	*k* = −7→7
1299 independent reflections	*l* = −17→15

### Refinement

**Table:**

Refinement on *F*^2^	Secondary atom site location: difference Fourier map
Least-squares matrix: full	Hydrogen site location: inferred from neighbouring sites
*R*[*F*^2^ > 2σ(*F*^2^)] = 0.034	H-atom parameters constrained
*wR*(*F*^2^) = 0.099	*w* = 1/[σ^2^(*F*_o_^2^) + (0.0722*P*)^2^ + 0.0677*P*] where *P* = (*F*_o_^2^ + 2*F*_c_^2^)/3
*S* = 1.05	(Δ/σ)_max_ = 0.001
1299 reflections	Δρ_max_ = 0.16 e Å^−^^3^
182 parameters	Δρ_min_ = −0.11 e Å^−^^3^
1 restraint	Absolute structure: 768 Friedel pairs merged
Primary atom site location: structure-invariant direct methods	

### Fractional atomic coordinates and isotropic or equivalent isotropic displacement parameters (Å^2^)

**Table:**

	*x*	*y*	*z*	*U*_iso_*/*U*_eq_	
O1	−0.00290 (12)	0.4999 (2)	0.24429 (14)	0.0590 (5)	
N1	0.0000	0.3969 (4)	0.0000	0.0453 (6)	
N2	0.00872 (13)	0.1951 (3)	0.18302 (16)	0.0503 (5)	
C1	0.0000	0.8221 (5)	0.0000	0.0582 (8)	
H1	0.0000	0.9632	0.0000	0.070*	
C2	−0.00113 (15)	0.7152 (4)	0.0817 (2)	0.0538 (6)	
H2	−0.0023	0.7831	0.1377	0.065*	
C3	−0.00049 (13)	0.5041 (3)	0.07933 (18)	0.0449 (5)	
C4	0.00168 (14)	0.3864 (4)	0.16940 (19)	0.0472 (5)	
C5	0.00709 (15)	0.1499 (4)	0.2830 (2)	0.0505 (5)	
H5	0.0604	0.0679	0.3445	0.061*	
C6	−0.07812 (16)	0.0392 (4)	0.2397 (2)	0.0522 (5)	
C7	−0.16168 (18)	0.0935 (5)	0.1346 (2)	0.0769 (8)	
H7	−0.1661	0.1950	0.0859	0.092*	
C8	−0.2399 (2)	−0.0029 (6)	0.1005 (3)	0.0909 (10)	
H8	−0.2964	0.0356	0.0294	0.109*	
C9	−0.2346 (2)	−0.1521 (6)	0.1699 (4)	0.0887 (10)	
H9	−0.2872	−0.2154	0.1468	0.106*	
C10	−0.1519 (2)	−0.2093 (5)	0.2738 (3)	0.0833 (9)	
H10	−0.1481	−0.3124	0.3212	0.100*	
C11	−0.07370 (18)	−0.1150 (4)	0.3090 (2)	0.0648 (7)	
H11	−0.0175	−0.1553	0.3800	0.078*	
C12	0.01565 (15)	0.3640 (4)	0.33649 (19)	0.0498 (5)	
H12	−0.0314	0.3795	0.3447	0.060*	
C13	0.10798 (15)	0.4089 (4)	0.45661 (19)	0.0493 (5)	
C14	0.14229 (19)	0.2821 (5)	0.5543 (2)	0.0654 (7)	
H14	0.1074	0.1742	0.5463	0.078*	
C15	0.2283 (2)	0.3148 (6)	0.6638 (3)	0.0788 (8)	
H15	0.2515	0.2268	0.7284	0.095*	
C16	0.27910 (19)	0.4766 (5)	0.6770 (3)	0.0738 (8)	
H16	0.3367	0.4995	0.7506	0.089*	
C17	0.24501 (16)	0.6032 (5)	0.5823 (3)	0.0712 (8)	
H17	0.2794	0.7136	0.5916	0.085*	
C18	0.16001 (17)	0.5710 (4)	0.4720 (2)	0.0600 (6)	
H18	0.1378	0.6592	0.4079	0.072*	

### Atomic displacement parameters (Å^2^)

**Table:**

	*U*^11^	*U*^22^	*U*^33^	*U*^12^	*U*^13^	*U*^23^
O1	0.0850 (11)	0.0442 (10)	0.0564 (9)	0.0107 (8)	0.0489 (9)	0.0016 (8)
N1	0.0524 (14)	0.0389 (14)	0.0499 (14)	0.000	0.0350 (12)	0.000
N2	0.0666 (11)	0.0425 (12)	0.0597 (11)	−0.0016 (9)	0.0486 (10)	−0.0038 (9)
C1	0.070 (2)	0.0344 (17)	0.061 (2)	0.000	0.0377 (17)	0.000
C2	0.0642 (14)	0.0395 (13)	0.0570 (13)	0.0013 (11)	0.0381 (12)	−0.0048 (11)
C3	0.0466 (12)	0.0397 (13)	0.0473 (11)	0.0004 (9)	0.0294 (10)	−0.0013 (9)
C4	0.0525 (12)	0.0443 (13)	0.0496 (12)	0.0032 (10)	0.0347 (10)	−0.0006 (10)
C5	0.0618 (13)	0.0449 (13)	0.0549 (12)	−0.0013 (10)	0.0418 (11)	−0.0010 (11)
C6	0.0666 (13)	0.0445 (12)	0.0565 (12)	−0.0065 (11)	0.0442 (12)	−0.0076 (10)
C7	0.0767 (17)	0.079 (2)	0.0661 (15)	−0.0148 (16)	0.0411 (15)	0.0034 (15)
C8	0.0714 (18)	0.103 (3)	0.0825 (19)	−0.0200 (19)	0.0413 (16)	−0.010 (2)
C9	0.090 (2)	0.083 (2)	0.117 (2)	−0.0336 (19)	0.078 (2)	−0.029 (2)
C10	0.106 (2)	0.0622 (18)	0.122 (2)	−0.0105 (17)	0.092 (2)	0.0006 (19)
C11	0.0817 (16)	0.0521 (14)	0.0830 (17)	0.0032 (13)	0.0629 (15)	0.0058 (14)
C12	0.0648 (13)	0.0450 (13)	0.0543 (12)	0.0017 (11)	0.0448 (12)	−0.0005 (10)
C13	0.0642 (13)	0.0468 (13)	0.0546 (12)	−0.0019 (10)	0.0460 (11)	−0.0041 (10)
C14	0.0837 (17)	0.0614 (17)	0.0612 (14)	−0.0122 (14)	0.0507 (14)	−0.0017 (13)
C15	0.0943 (19)	0.080 (2)	0.0591 (15)	0.0159 (19)	0.0474 (15)	0.0108 (16)
C16	0.0644 (15)	0.078 (2)	0.0718 (16)	0.0007 (15)	0.0399 (14)	−0.0115 (16)
C17	0.0644 (16)	0.0689 (18)	0.0885 (18)	−0.0091 (14)	0.0523 (16)	−0.0086 (16)
C18	0.0687 (15)	0.0554 (15)	0.0696 (14)	−0.0040 (13)	0.0504 (13)	0.0000 (12)

### Geometric parameters (Å, °)

**Table:**

O1—C4	1.354 (3)	C8—H8	0.9300
O1—C12	1.440 (3)	C9—C10	1.362 (5)
N1—C3	1.341 (2)	C9—H9	0.9300
N1—C3^i^	1.341 (2)	C10—C11	1.379 (4)
N2—C4	1.270 (3)	C10—H10	0.9300
N2—C5	1.480 (3)	C11—H11	0.9300
C1—C2	1.376 (3)	C12—C13	1.513 (3)
C1—C2^i^	1.376 (3)	C12—H12	0.9800
C1—H1	0.9300	C13—C18	1.376 (3)
C2—C3	1.392 (3)	C13—C14	1.384 (3)
C2—H2	0.9300	C14—C15	1.386 (4)
C3—C4	1.481 (3)	C14—H14	0.9300
C5—C6	1.507 (3)	C15—C16	1.370 (5)
C5—C12	1.563 (3)	C15—H15	0.9300
C5—H5	0.9800	C16—C17	1.355 (4)
C6—C7	1.371 (4)	C16—H16	0.9300
C6—C11	1.384 (3)	C17—C18	1.381 (4)
C7—C8	1.392 (4)	C17—H17	0.9300
C7—H7	0.9300	C18—H18	0.9300
C8—C9	1.355 (5)		
			
C4—O1—C12	106.19 (17)	C10—C9—H9	120.1
C3—N1—C3^i^	116.5 (2)	C9—C10—C11	120.4 (3)
C4—N2—C5	106.44 (19)	C9—C10—H10	119.8
C2—C1—C2^i^	118.4 (3)	C11—C10—H10	119.8
C2—C1—H1	120.8	C10—C11—C6	120.6 (3)
C2^i^—C1—H1	120.8	C10—C11—H11	119.7
C1—C2—C3	119.1 (2)	C6—C11—H11	119.7
C1—C2—H2	120.5	O1—C12—C13	110.46 (18)
C3—C2—H2	120.5	O1—C12—C5	103.00 (15)
N1—C3—C2	123.5 (2)	C13—C12—C5	114.64 (19)
N1—C3—C4	116.65 (18)	O1—C12—H12	109.5
C2—C3—C4	119.9 (2)	C13—C12—H12	109.5
N2—C4—O1	118.7 (2)	C5—C12—H12	109.5
N2—C4—C3	126.5 (2)	C18—C13—C14	118.5 (2)
O1—C4—C3	114.73 (18)	C18—C13—C12	122.2 (2)
N2—C5—C6	112.22 (19)	C14—C13—C12	119.3 (2)
N2—C5—C12	103.37 (18)	C13—C14—C15	120.5 (3)
C6—C5—C12	112.88 (18)	C13—C14—H14	119.7
N2—C5—H5	109.4	C15—C14—H14	119.7
C6—C5—H5	109.4	C16—C15—C14	120.0 (3)
C12—C5—H5	109.4	C16—C15—H15	120.0
C7—C6—C11	118.5 (2)	C14—C15—H15	120.0
C7—C6—C5	121.4 (2)	C17—C16—C15	119.6 (3)
C11—C6—C5	120.1 (2)	C17—C16—H16	120.2
C6—C7—C8	120.2 (3)	C15—C16—H16	120.2
C6—C7—H7	119.9	C16—C17—C18	121.1 (3)
C8—C7—H7	119.9	C16—C17—H17	119.5
C9—C8—C7	120.6 (3)	C18—C17—H17	119.5
C9—C8—H8	119.7	C13—C18—C17	120.3 (3)
C7—C8—H8	119.7	C13—C18—H18	119.9
C8—C9—C10	119.7 (3)	C17—C18—H18	119.9
C8—C9—H9	120.1		
			
C2^i^—C1—C2—C3	0.42 (15)	C8—C9—C10—C11	0.5 (5)
C3^i^—N1—C3—C2	0.45 (17)	C9—C10—C11—C6	0.1 (5)
C3^i^—N1—C3—C4	−178.3 (2)	C7—C6—C11—C10	−1.0 (4)
C1—C2—C3—N1	−0.9 (3)	C5—C6—C11—C10	176.2 (3)
C1—C2—C3—C4	177.79 (15)	C4—O1—C12—C13	109.2 (2)
C5—N2—C4—O1	1.7 (3)	C4—O1—C12—C5	−13.6 (2)
C5—N2—C4—C3	−179.57 (19)	N2—C5—C12—O1	14.4 (2)
C12—O1—C4—N2	8.5 (3)	C6—C5—C12—O1	−107.09 (19)
C12—O1—C4—C3	−170.44 (17)	N2—C5—C12—C13	−105.65 (19)
N1—C3—C4—N2	3.9 (3)	C6—C5—C12—C13	132.9 (2)
C2—C3—C4—N2	−174.8 (2)	O1—C12—C13—C18	5.3 (3)
N1—C3—C4—O1	−177.25 (15)	C5—C12—C13—C18	121.1 (2)
C2—C3—C4—O1	4.0 (3)	O1—C12—C13—C14	−173.81 (18)
C4—N2—C5—C6	111.8 (2)	C5—C12—C13—C14	−58.0 (3)
C4—N2—C5—C12	−10.1 (3)	C18—C13—C14—C15	−1.9 (4)
N2—C5—C6—C7	−42.1 (3)	C12—C13—C14—C15	177.2 (2)
C12—C5—C6—C7	74.2 (3)	C13—C14—C15—C16	1.7 (4)
N2—C5—C6—C11	140.8 (2)	C14—C15—C16—C17	−0.4 (4)
C12—C5—C6—C11	−102.8 (2)	C15—C16—C17—C18	−0.5 (4)
C11—C6—C7—C8	1.1 (4)	C14—C13—C18—C17	1.0 (3)
C5—C6—C7—C8	−176.0 (3)	C12—C13—C18—C17	−178.2 (2)
C6—C7—C8—C9	−0.5 (5)	C16—C17—C18—C13	0.3 (4)
C7—C8—C9—C10	−0.3 (5)		

Symmetry codes: (i) −*x*, *y*, −*z*.
